# Gender Affirmation and Resiliency Among Black Transgender Women With and Without HIV Infection

**DOI:** 10.1089/trgh.2016.0005

**Published:** 2016-05-01

**Authors:** Richard A. Crosby, Laura F. Salazar, Brandon J. Hill

**Affiliations:** ^1^ Kinsey Institute for Research on Sex, Gender, and Reproduction, Indiana University, Bloomington, Indiana.; ^2^ College of Public Health, University of Kentucky, Lexington, Kentucky.; ^3^ Department of Health Promotion and Behavior, School of Public Health, Georgia State University, Atlanta, Georgia.; ^4^ Center for Interdisciplinary Inquiry and Innovation in Sexual and Reproductive Health, Department of Obstetrics and Gynecology, University of Chicago, Chicago, Illinois.

**Keywords:** black, gender affirmation, HIV infections, transgender women

## Abstract

**Purpose:** Among black transgender women (transwomen) at high risk of HIV acquisition or already living with HIV/AIDS, the study examined whether medical or socially based gender affirming factors may contribute differentially to selected measures of resiliency, perceived stress, and a scale measure of mental health outcomes. This question has implications for clinical care and counseling of this population.

**Methods:** Seventy-seven black transwomen were recruited to participate in a private, face-to-face structured interview. Two index measures of gender affirmation (GA) were constructed from the data. One comprised medical aspects only and the other comprised social aspects of GA. Assessed outcomes were personal competence and acceptance of self and life (resiliency), perceived stress and recent anxiety, depression, and suicide ideation (mental health). Associations between GA variables and outcomes were first assessed using bivariate level correlations. Significant bivariate associations were then tested in multivariable regression models adjusting for age and HIV status.

**Results:** Mean age of the sample was 34.5 years. More than one-half of the sample (62.3%) indicated being HIV-infected. None of the bivariate or multivariable associations pertaining to GA medical factors were significant. Conversely, the social GA factors were significant and protective with all four outcomes. In the presence of age and HIV status, greater social GA was significantly associated with greater personal competence, acceptance of self and life, and positive mental health outcome. HIV status had an independent effect on personal competence, acceptance of self and life, with HIV-positive transwomen scoring higher on both measures.

**Conclusion:** Among black transwomen at high risk of HIV acquisition or already HIV-infected, study findings suggest the possibility that socially based GA may play a prominent role in strengthening the resiliency and mental health of black transwomen. This same protective effect may not occur as a consequence of gender affirming body modification practices.

## Introduction

As applied to transgender women, those assigned male at birth, but identify as women, female, or on the male-to-female spectrum, the concept of gender affirmation (GA) suggests that those who have higher satisfaction with their gender presentation and expression may have increased protection against stigma (internalized and experienced), discrimination, and transphobia, all of which marginalize and predispose this population to high-risk behaviors (e.g., sex work, condomless anal sex) and contribute to psychosocial morbidities.^1^ GA is described as perceiving validation of one's gender identity and expression, and it may involve having a body image that is concordant with one's gender identity, along with social recognition and legitimacy, and acceptance of the transgender-self.^2–5^ Thus, sources of GA are varied, and may include satisfaction with one's gender presentation, for example, physical appearance, and nonphysical psychosocial aspects relative to transgender identity, expression, and lived gender experience, for example, social transition, intimate and romantic relationships, and support.

For a number of transwomen, presenting a feminine or female-typical physical appearance has been associated with greater satisfaction with their gender identity and expression.^1,2,6–8^ Gender reassignment surgery, breast augmentation, facial feminization surgery (FFS), hormone replacement therapy (HRT), and other medical procedures may contribute to a physical presentation consistent with transwomen's gender identity and expression, and may be highly affirming for those who undergo medical transition. In turn, high satisfaction with gender expression may foster increased social acceptance and, consequently, alleviate distress associated with gender identity and body incongruence, ultimately improving the mental health of transgender people. For example, longitudinal data from transgender patients on cross-sex HRT suggest that HRT decreases psychiatric distress and improves mental health outcomes.^9^ Additionally, another study demonstrated that transwomen who had FFS had significantly higher levels of positive mental health, compared to transwomen who did not have FFS.^10^ Indeed, the current recommendations for treating transgender individuals focuses on ameliorating the distress associated with incongruency in assigned birth sex and gender identity.^11–13^

However, in addition to medical transition there are several aspects of social transition that can be gender affirming, including concordant gender identity, legal name, and sex marker (e.g., male/female) on state and federal identification documents. Discordant identification documents expose transgender people to stigma and discrimination each time identification is required (e.g., healthcare check-in, banking, travel, etc.). In contrast, concordant identification documents are a critical step in gaining social inclusion and legal legitimacy, which may have downstream effects on transgender health—particularly among those for whom access to medical transition might be limited due to resources (e.g., low-income transwomen). For example, in a cross-sectional study Hill et al. found that low-income transwomen of color who had changed their legal name on identification documents were significantly less likely to report delayed medical care, overnight hospitalization, and the use of nonmedically prescribed injectable hormones, compared to transwomen who had not changed their identification documents.^4^

Additionally, meaningful social and romantic relationships may provide transwomen a resource of social support and GA.^14^ For example, a qualitative study of transgender GA by Aguayo-Romero et al. found that social peers, including sexual and romantic partners of transwomen, constitute an important source of encouragement and gender identity acceptance.^15^ However, as Nuttbrock et al. have highlighted, GA through sexual relationships, including commercial sexual relationships (i.e., sex work), may have mental health benefits but may need to be balanced by potential health risks, including HIV risk.^14^ Further, Bockting et al. have suggested a gender affirming benefit for transwomen engaging in receptive anal sex.^16^

Regardless of source, GA among transgender people has been associated with a host of positive mental health outcomes, including increased resilience, improved coping with stress, and positive emotional affect^17–19^; however, little is known about whether these relationships apply to populations of black transwomen at high risk of HIV acquisition or already living with HIV/AIDS. Given that transwomen, particularly transwomen of color, remain disproportionately burdened by the HIV epidemic in the United States,^20–22^ understanding the role of GA in this high-risk population is significant. It is plausible that living with HIV may be an “all encompassing” part of life and daily stress and stigma, minimizing the positive effects of GA. However, if the benefits of GA for HIV-positive transwomen of color are not met in the same manner as their HIV-negative counterparts, additional support and counseling interventions and therapy programs may be warranted to take full advantage of the protective value of GA.^1^

A primary research question that remains unexplored in the published literature involves GA and the mental health of black transwomen at risk of HIV acquisition or HIV transmission. The question of whether medical aspects of GA (gender affirming medical transition) or social factors (e.g., legal name change, meaningful romantic or sexual relationships, greater satisfaction with gender identity) may be equally important. Accordingly, the purpose of this study involving black transwomen at risk of HIV acquisition or HIV transmission was as follows: (1) to examine whether medical or socially based gender affirming factors contribute to positive mental health outcomes (e.g., resilience, coping with stress), and (2) to examine whether HIV-status is independently associated with the same mental health outcomes. These research questions are important because the answers have direct implications for the counseling and clinical practices of providers serving populations of black transwomen.

## Methods

### Participants and procedures

Multiple community-based outreach strategies were used to recruit a community sample of black transwomen between ages 18 and 65 years, residing in Atlanta, GA. Venues serving transgender women and word of mouth from transgender advocates served as the primary methods for recruitment. The study was known as the “Transgender Atlanta Personal Survey (TAPS)” and it was designed to provide an initial data source pertaining to the overall health and well-being of black transwomen at high risk of HIV acquisition or living with HIV/AIDS. Transgender advocates notified the study project director when they identified an eligible transgender woman willing to be screened for study participation. Additionally, the project was advertised through formal and informal communication channels via advocacy groups and lesbian, gay, bisexual, and transgender (LGBT) service organizations. Print materials provided contact information for the project director. All data were collected from October 2014 to June 2015.

All participants were screened to determine eligibility. Requirements included the following: (1) being 18 to 65 years of age; (2) having been assigned male at birth and self-identifying as either a transgender woman, female, or other gender-nonconforming identity; and (3) reporting anal sex with a cisgender (non-transgender) male in the past 6 months. After providing written informed consent, participants were engaged in a face-to-face structured interview with a trained graduate research assistant lasting ∼60–90 minutes. All interview survey responses were recorded on a portable electronic tablet using Qualtrics© software (Provo, UT). The Institutional Review Board for research on human subjects at Georgia State University approved all study protocols and procedures.

### Measures

#### Sociodemographic characteristics

Sociodemographic characteristics assessed age, race, self-reported HIV-status, education, employment, and income.

#### Medically based gender affirming factors

An index of medically based GA factors comprised six items, including the following: (1) having had sex/gender reassignment (assessed by a single questionnaire item, “Have you had sex reassignment surgery?”); (2) breast implants/augmentation; (3) take or have taken hormones; (4) having had surgically altered the body; (5) currently receiving HRT under the supervision of a healthcare provider; and (6) having ever injected, or been injected, with a substance such as silicone to change the shape of the body. Response options were forced choice “yes”/“no” (Table 1).

**Table 1.** **Factors Comprising the Two Gender Affirmation Index Measures and the Number of Participants Indicating “Yes” to Each Item**

	Number (%) “Yes”
Medically based gender affirmation factors	
Have you had sex reassignment surgery?	7 (9.1)
Do you have breast implants?	13 (16.9)
I take/have taken hormones	61 (79.2)
I have surgically altered my body	17 (22.1)
Are you currently receiving hormone therapy under the supervision of a healthcare provider	32 (41.6)
Used hormones orally, topically, or injected–past 12 months^a^	15 (19.5)
Have you ever injected, or been injected, with a substance such as silicone to change the shape of your body?	22 (28.6)
Social gender affirmation factors	
Have you legally changed your name to better reflect your gender identity?	34 (44.2)
Does a legal photo ID (such as a driver's license) show that you are female?	13 (16.9)
Currently in a relationship with someone you consider to be a boyfriend/girlfriend, partner, or significant other	33 (42.9)
Disagree with statement, “I avoid thinking about my gender identity”	31 (40.3)
Generally find sex to be “blissful”	44 (57.1)

^a^ This item was only counted for those women not counted as “yes” for the previous item.

#### Social gender affirming factors

Five items were used to assess social gender affirming factors, including the following: (1) having legally changed their name to reflect gender identity; (2) having a legal photo ID with a female sex indication; (3) disagreeing with avoidance of thinking about gender identity; (4) currently in a relationship with a boyfriend/girlfriend, partner, or significant other; and (5) reporting finding sex to be “blissful.” The inclusion of the latter two items of relationship and sexual pleasure reflect more dyadic dimensions to GA,^23,24^ typically absent in the research literature on GA (Table 1).

#### Resilience

The Wagnild and Young Resilience Scale,^25^ was used to assess resilience. Response options included a 7-point Likert scale, 1=strongly disagree, 7=strongly agree. The 25-item scale was reduced to two 6-item subscales. Using factor analysis the factor loadings of 0.70 or greater constituted the first 6-item subscale generally reflecting *personal competence* in everyday life (Table 2). Inter-item reliability was excellent with a Cronbach's alpha=0.87. The second subscale, *acceptance of self and life*, was composed of 6-items with factor loadings greater than 0.65. Inter-item reliability was good with a Cronbach's alpha=0.79.

**Table 2.** **Items Included in the Four Scale Measures**

	Mean (standard deviation)
Measure 1: Personal competence^a^	
I usually manage one way or another	6.25 (1.1)
I feel proud I have accomplished things in life	6.39 (1.1)
I am friends with myself	6.40 (0.81)
I have self-discipline	6.14 (1.2)
I can usually find something to laugh about	6.36 (0.72)
My life has meaning	6.49 (0.75)
Measure 2: Acceptance of self and life^a^	
Keeping interested in things is important to me	6.46 (0.78)
I usually take things in stride	6.06 (1.17)
I keep interested in things	6.12 (1.15)
I can usually look at a situation in a number of different ways	6.25 (0.90)
I am able to depend on myself more than anyone	6.49 (0.79)
I take things one day at a time	6.23 (1.13)
Measure 3: Perceived stress^b^ In the last month, how often have you:	
Been upset because of something that happened unexpectedly	2.96 (0.99)
Felt that you were unable to control important things in your life	2.70 (1.11)
Felt nervous or stressed	3.30 (1.08)
Found that you could not cope with all things you had to do	2.45 (1.06)
Been angered because of things outside of your control	3.03 (1.10)
Felt difficulties were piling up so high you could not overcome these	2.66 (1.07)
Been able to control irritations in life^c^	3.51 (1.01)
Felt you were on top of things^c^	3.62 (1.12)
Measure 4: Depression, anxiety, and suicidal ideation^b^ How much have you felt the following in the past 7 days?	
Feeling not interested in things	2.11 (1.14)
Feeling lonely	2.64 (1.44)
Feeling blue	2.42 (1.31)
Feeling worthless	1.67 (1.11)
Feeling hopeless about the future	1.93 (1.28)
Thoughts about ending your life	1.11 (0.52)
Nervousness or shakiness inside	2.03 (1.21)
Suddenly scared for no reason	1.59 (1.10)
Spells of terror or panic	1.50 (1.00)
Feeling fearful	1.70 (1.01)

^a^ Response options provided on a 7-point Likert scale with higher numbers equating with greater levels of the construct.

^b^ Response options provided on a 5-point Likert scale with higher numbers equating with greater levels of the construct.

^c^ Reverse coded.

#### Perceived stress

The Perceived Stress Scale (Sheldon) was used to assess perceived social stress within the last month. Response options included a 5-point Likert scale, 0=never, 4=very often.^26^ For this sample the 10-item scale was reduced to 8 items (Table 2) to obtain adequate inter-item reliability (Cronbach's alpha=0.75).

#### Mental health outcomes

The Brief Symptom Inventory was used to assess symptoms of anxiety, depression, and suicidal ideation over the past 7 days.^27^ Response options included a 5-point Likert scale, 0=not at all, 4=extremely. This scale was reduced to a 10-item measure to achieve a Cronbach's alpha of 0.88 (Table 2).

#### HIV status

As a covariate we assessed women's HIV status with a single item: “What was the result of your most recent HIV test?” Women never having a test for HIV and those indicating “negative” were counted as not knowingly HIV-infected, with the remainder being counted as knowingly HIV-infected.

### Data analysis

A 6-item index of medically based GA factors and a 5-item index of social GA factors served as the two primary correlates. These measures formed normal distributions and thus were preserved in their continuous forms. The four scale measures also formed relatively normal distributions and were thus treated as continuous variables. Bivariate associations between each index measure and each scale measure were assessed using Pearson Product Moment Coefficients. Subsequently, multiple linear regression models were used to generate age and HIV-status adjusted estimates of associations between the index measures and the mental health scale measures. Significance was defined by a *p*-value of <0.05.

## Results

### Characteristics of the sample

The sample of 77 transwomen ranged in age from 18 to 65 years of age (mean=34.5; standard deviation=10.6). All participants identified as black or African American. More than one-half of the sample (62.3%) indicated being HIV-infected, with 35.1% indicating their last test result was negative, one transwoman indicating she did know her HIV status, and one choosing not to answer this question. Completing not more than high school education was reported by 54.5% of the women. Full-time employment was indicated by 20.8% and part-time employment was indicated by 11.7%. The majority (61.0%) indicated their annual income was less than $10,000, with 13.0% indicating annual incomes greater than $30,000.

The mean score on the index assessing *medically based* aspects of GA was 2.17 (SD=1.55), with a range of 0 to 6. The mean score on the index assessing *social* aspects of GA was 2.01 (SD=1.28), with a range of 0 to 4.

The mean score on the scale measure labeled “personal competence” was 38.0 (SD=4.5), with a range of 15 to 42 (possible range=15–42). The mean score on the scale measure labeled “acceptance of self and life” was 37.5 (SD=4.2), with a range of 20 to 42 (possible range=1–42). The mean score on the scale measure labeled “perceived stress” was 21.97 (SD=5.2), with a range of 9 to 35 (possible range=8–40). Finally, the mean score on the scale measure labeled “mental health outcomes” was 18.7 (SD=7.8), with a range of 10 to 50 (possible range=10–50).

### Bivariate associations

Table 3 displays the observed bivariate associations. As shown, none of the correlation coefficients pertaining to the 6-item index of GA medically based factors were significant. Conversely, all four coefficients pertaining to the 5-item index of GA social factors were significant and strong (with three exceeding 0.30) with directions suggestive of being protective.

**Table 3.** **Observed Bivariate Associations (Pearson Correlation Coefficients) Between Index Measures and the Four Outcome Measures**

Outcome measure	Medical index, *r* (*p*)	Social index, *r* (*p*)
Personal competence	−0.01 (0.91)	0.378 (0.001)
Acceptance of self and life	0.03 (0.77)	0.365 (0.001)
Perceived stress	0.04 (0.73)	−0.251 (0.03)
Depression, anxiety, and suicidal ideation	−0.11 (0.33)	−0.337 (0.003)

### Multivariate associations

Table 4 displays the age and HIV-status adjusted Beta estimates for associations between the 5-item index of GA social factors and the four outcome measures. As shown, in the presence of age and HIV status, three of these four outcomes maintained a significant association with the index measure. One outcome (daily stress) did not retain significance in the adjusted model. Also as shown, HIV status had an independent effect on both resilience subscales. As coded, the positive Beta estimates indicated that HIV-infected women had significantly greater levels of resilience.

**Table 4.** **Age and HIV-Status Adjusted Beta Estimates for Models Pertaining to the Index of Socially Based Gender Affirmation Factors**

	Age, β (*p*)	HIV status,^a^ β (*p*)	5-Item index, β (*p*)
Personal competence	−0.31 (0.009)	0.25 (0.02)	0.50 (<0.001)
Acceptance of self and life	−0.26 (0.03)	0.26 (0.02)	0.46 (<0.001)
Perceived stress	−0.14 (0.27)	−0.06 (0.60)	−0.19 (0.13)
Mental health outcomes	−0.10 (0.44)	−0.08 (0.47)	−0.29 (0.02)

^a^ This variable was coded as “0” for not knowingly HIV-infected and “1” for HIV-infected.

## Discussion

The first purpose of this study was to examine whether medical or socially based gender affirming factors contribute to positive mental health outcomes (e.g., resilience, coping with stress). The findings were unequivocal and support the strong association of social gender affirming factors with three of four selected mental health outcomes. The findings were also unequivocally in support of the concept that medically based GA factors are not associated with positive mental health outcomes such as resilience, coping with stress, and positive affect. Together, these distinct sets of findings suggest that social and structural factors (e.g., legal name change and legal identification documents) and relational factors (e.g., meaningful relationships and “blissful sex”) may serve as beneficial focal points for GA efforts aimed at increasing resilience in the day-to-day lives of black transwomen. Fostering resilience may be especially important for this population given the historical significance of poverty, sexism, and transphobia experienced by black transwomen in the United States.^28,29^

The second purpose of the study was to determine whether living with HIV is independently associated with four indicators of mental health. Indeed, this was the case with respect to both resilience subscales (Personal Competence and Acceptance of Self and Life). In both cases the observed standardized Beta estimate was substantial and significant. Perhaps contrary to what might be expected, however, the direction of these associations suggests that women living with HIV have relatively greater levels of resilience than their HIV-uninfected counterparts. The observation that this effect was independent from the influence of GA is important because it implies that black transwomen living with HIV may be highly resilient. Whether this apparent protective effect of living with HIV is a product of the “warp-around” services provided through the Ryan White Care Act or the host of social services available to individuals who are HIV-positive (e.g., disability coverage, social security benefits, housing, etc.) is a valid question for future research.^30^ The Ryan White Care Act (a congressional mandate to provide comprehensive HIV/AIDS care) has been instrumental in the United States relative to improving the quality of life for those living with HIV/AIDS.

Several implications can be derived from our findings. First, the null findings relative to the medical index of GA can potentially be viewed through the lens of body objectification theory.^31^ Previous research suggest that transwomen, like non-transgender (cisgender) women, may view themselves as sexual objects and thus engage in body monitoring and corresponding feelings of physical inadequacy.^7,32^ Objectification of the body may become a never-ending source of stress and anxiety. In our sample of black transwomen, the consistent null findings suggest that body modification practices may not be as important as the nonphysical factors to the four constructs we assessed. However, the majority of transwomen in this study reported having low income, which may also determine the degree to which transwomen may have the resources to utilize medically based gender affirming procedures. From a clinical perspective, gender affirming psychotherapy may be far more valuable to the mental health of black transwomen than gender affirming body modification procedures.

A second implication involves the fairly robust findings relative to the 5-item index of social gender affirming factors. This index is relatively unique in that it includes two items not typically assessed in GA: (1) being in a meaningful relationship and (2) reporting that sex is generally “blissful.” The relatively greater levels of resilience and positive affect experienced by transwomen scoring higher on the index suggests that romantic or sexual relationships and sexual satisfaction may be potentially equally gender affirming as larger social aspects for example, legal name change and photo identification showing female sex/gender status. Future research as to how attitudes/skills can be fostered in black transwomen that help them enjoy sex may indeed have multiple benefits to their overall mental health. Research studies of cisgender females has suggested strong associations between sexual satisfaction and mental health outcomes.^33^

A third and final implication involves the basic descriptive findings relative to the four measures of mental health. For each scale the observed means suggest overall high levels of resilience, coping ability, and positive affect. Given that more than 60% of the women were living with HIV these means clearly suggest that black transwomen (at least those volunteering for study participation) may be a resilient population from a mental health perspective. This is an important possibility to consider given the multiple forms of stigma, discrimination, and prejudice experienced by black transwomen.^34^ Clinically, this suggestion implies that therapeutic strategies can be constructed using a strength building perspective or asset-based models of GA.^35–37^ In particular, interventions aimed at increasing self-esteem and transgender pride and group identity using the GA framework may benefit by leveraging black transwomen's existing resilience, coping abilities, and positive affect.^36,38^ Additionally, these findings support previous research underscoring the importance of more holistic approaches to transgender GA, including integrating GA as part of community assessment, research, education, training, and advocacy alongside clinical care.^38,39^ Lastly, our findings of relatively good mental health also demonstrate the benefit of asset-based approaches to transgender health interventions, rather than deficit models that tend to focus on shortcomings or inadequacies (e.g., health disparities, individual-level risk behaviors, etc.). Asset-based approaches emphasize strengths and individual-level self-management of transgender persons, even in light of social marginalization and injustice.^37,39^

### Limitations

The majority of participants were recruited through referrals from community-based organizations providing services and support to transwomen. Whether these women are representative of black transwomen not receiving similar support and services is not known. Also, several measurement-related limitations apply. For example, factor analysis can produce an infinite number of possible solutions therefore definitive subscales are not possible. That the two subscales derived from the larger 25-item measure of resilience were constructed and used in the same sample is a potential limitation. Although not a limitation *per se*, it is worth noting that the measure of living with HIV was not entirely accurate as it compared those who were knowingly infected to those not knowingly infected; however, the latter group comprised women having recent HIV tests and women never being tested. Finally, it is noteworthy that the significant bivariate association between the 5-item index of socially based gender affirming factors and the scale measure of daily stress did not retain significance in the linear regression model. This occurred despite both age and HIV status also being non-significant. This may have been due to a lack of adequate statistical power.

## Conclusion

Study finding suggest the possibility that socially based gender affirming factors may play a prominent role is strengthening the resilience and positive affect of black transwomen, regardless of age or HIV status. Moreover, this same protective effect may not occur as a consequence of gender affirming body modification practices and procedures. Lastly, black transwomen living with HIV may be experiencing improved resilience as a consequence of yet to be identified aspect of wrap-around services, included in the Ryan White Care Act or the host of social service resources available to transwomen who are HIV-positive compared to those who are HIV-negative (e.g., disability coverage, housing assistance, etc.).

## Abbreviations Used

FFS: facial feminization surgery
GA: gender affirmation
HRT: hormone replacement therapy
